# Design Authorship: an intrinsic driver of designer-entrepreneurs

**DOI:** 10.12688/openreseurope.14148.1

**Published:** 2021-11-02

**Authors:** Aldo Valencia, Alison Pearce, Martin Ryan

**Affiliations:** 1 Design Innovation, National University of Ireland, Maynooth, Maynooth, Kildare, W23PR94, Ireland; 2 Newcastle Business School, Northumbria University, Newcastle, Tyne and Wear, NE1 8ST, UK; 3 Design, National College of Art and Design, Dublin, Dublin, D08 K521, Ireland

**Keywords:** Design Entrepreneurship, Product start-up, Design Innovation, Design Authorship, Business Innovation.

## Abstract

This paper describes the entrepreneurial journey of product designers and the driver that makes them take an idea into the market. Following a Constructivist Grounded Theory approach, a multiple-phase data generation method explored the entrepreneurial journey of eleven designer-entrepreneurs (D-entrepreneurs). The paper describes the driver named design authorship (D-authorship) and why it is essential in the entrepreneurial journey of designers. The study identified two types of D-authorship: a) the inside-out, where D-entrepreneurs spent considerable time obtaining perfection in the product without any user feedback involved, and b) the outside-in, where D-entrepreneurs build their product as a result of a systematic user-centric approach.

## Plain language summary

Product designers are equipped with the right skills and knowledge to create new products. This paper describes the entrepreneurial journey of eleven designer-entrepreneurs starting their product-based companies. However, most of these products never reach the market because of the gap between invention and product launch. This research has identified a critical driver that made these designers go the extra mile and finally take their products to the marketplace; we call it Design Authorship (D-authorship). The study identified two types of D-authorship: a) the inside-out, where D-entrepreneurs spent considerable time obtaining perfection in the product without any user feedback involved, and b) the outside-in, where D-entrepreneurs build their product as a result of a systematic user-centric approach.

## Introduction

Design thinking has proven its potential to understand the customer’s needs, accelerate time to market and reduce the risk of failure, without neglecting aesthetics and customer experience, in business settings ( Best, 2011; Borja de Mazota, 2003; Brown, 2008; Design Council, 2018; Ries, 2011). Businesses incorporate design thinking into their teams because it can act as a bridge between different disciplines and their final users ( Kelley & VanPatter, 2005) and enhance business performance. Designers tap into empathic tools like personas in order to bring an empathetic compass to the product development process. It has been proven that the use of personas is an effective catalyst for innovation ( So & Joo, 2017). According to Dorst (2011, 522) designers have been dealing with “open, complex problems for so many years” that they have developed professional ways to handle them that could be useful for organisations. The design landscape has recently seen a new type of role emerge outside of corporate settings. More designers are increasingly becoming designer-entrepreneurs (D-entrepreneurs) ( Colombo *et al.*, 2017; Gaglione & Gaziulusoy, 2019; Mata García *et al.*, 2017) expanding the design discipline’s reach. Without business knowledge, design is handicapped to “influence the future directions in production systems driven by market forces” ( Teixeira, 2010, 417).

Multiple ways have been identified for entrepreneurs to identify opportunities. Lee *et al.* (2020) summarised six ways in which entrepreneurs identify opportunities: find jobs to be done, create mentor and social networks, pattern detection, apply prior knowledge, structural alignment, and hypothesis testing. The goal of design thinking is to identify opportunities from an empathic standpoint with the user at its centre. However, there is no evidence showing how D-entrepreneurs identify opportunities to start a business.

In this paper, we question whether user-centricity is a characteristic that prevails in the entrepreneurial journey of some designers. There are still presumptions about designers assuming the role of entrepreneurs that have been drawn from the traditional designer´s practice in the corporate setting.

 This paper calls into question the expectation that user centricity is used by designers in their entrepreneurial process. The main objectives are a) to identify the milestones´ sequence taken by designers in their entrepreneurial journey, b) to find whether user-centricity plays a significant role in the decisions made throughout the entrepreneurial journey of designers, and if user centricity is not relevant in their processes, and c) what is it that drive, inspire and guide them in the entrepreneurial journey.

### Literature review - design and entrepreneurship processes

Entrepreneurship encompasses the set of actions, mindsets and processes that enable individuals to turn an idea into a product capable of reaching the market ( Carayannis, 2013). Entrepreneurs focus on the value creation and establishment of new business ( Lou, 2015), while designers focus on the creation of new products, services, and new experiences that concern with what will exist and is unobservable ( Bonsiepe, 2007; Krippendorff, 2008). Both profiles overlap in their pursuit of novelty, value creation and impact.

To explain the uncommon behaviours of entrepreneurs, such as prolonged intense focus, unconventional risk-taking and unwavering belief in personal ideals, researchers have appealed to the concept of passion ( Cardon *et al.*, 2009). Passion arouses positive emotions in individuals, facilitating new information processing and stimulating the flow state of individuals that ultimately decreases the worry of failure and the awareness of time ( Dietrich, 2015). Cardon *et al.* (2009) conclude that positive emotions motivate entrepreneurs to tackle challenges in the entrepreneurial journey. Positive emotions can also be related to the cognitive ease ( Kahneman, 2011) experienced by individuals facing a new task. Cognitive ease refers to how easily the brain can process information without requiring extra attention or mental work. Therefore, passion is deemed as an important driver of entrepreneurs, facilitating the adaptability of performing new tasks and new challenges, influencing the motivation to continue the entrepreneurial journey.

Bleda, Querbes, and Healey (2021) studied the influence of motivational factors on ongoing product design decisions. They proved that better designs are achieved when designers are motivated by accomplishing a successful innovative design. Designers learn from their customers through empathy, the underpinning principle of user-centricity and customer feedback. Empathy is one of the main principles of Design Thinking ( Selloni & Corubolo, 2017); it connects the researcher, the user and its context, drawing a more thorough understanding of the problem.

“Design thinking is rooted in the principle that to design a great product or service, one must develop empathy for and deep insight into the customer’s behaviours and needs. Teams spend time with customers from the beginning of the development process, asking questions, rapidly generating multiple ideas, and testing them. The point is not to validate or prove an idea ‘right’, but to get instant, unfiltered reaction” ( Leichter, 2011, para. 3).

So & Joo (2017) proved that the use of personas (a proxy for the target audience based on user research) increases the originality of ideas in the ideation stage. Also, an empathic approach can help to overcome design fixation when there is a concise and consistent understanding of the user. The available literature on design innovation methods, such as the Design Council framework for innovation ( Design Council, 2019) and IDEO’s human-centred design toolkit, emphasises the importance of personas in the innovation process.

From the entrepreneurial perspective, empathy has been integrated into existing models of entrepreneurship as user research and customer feedback throughout the development cycle. Methods like lean start-up ( Ries, 2011), design venture and lean design thinking ( Müller & Thoring, 2012) claim the importance of the user centricity to increase the chances of business success.

***Design models.*** More than a hundred models for creativity, design, entrepreneurship and innovation have been catalogued by researchers ( Baregheh *et al.*, 2009; Howard *et al.*, 2008; VanPatter & Pastor, 2016), starting with the Helmholtz description of the creative process (1826) right up to the latest ‘design sprint’ ( Knapp *et al.*, 2016), ‘radical innovation of meanings’ ( Verganti, 2016) and the Design Council framework for innovation ( Design Council, 2019).

Howard *et al.* (2008) classified 23 unique design models, identifying six overarching general phases:

- Establishing the needs
- Analysis of task phase
- Conceptual design
- Embodiment design phase
- Detail design phase
- Production, use, retirement

VanPatter & Pastor (2016) classified the last 80 years of innovation methods, identifying four phases:

- Discover and orient
- Define and conceptualise
- Optimise and plan
- Execute and measure

These phases encompass creative problem-solving models and processes in product and service design, organisational and societal innovation. The phases in both classifications resemble to a corporate system, where designers depend on and interact with other disciplines and departments, having a specific role and semi-fixed set of activities. Therefore, designers working within a company cover particular stages of these innovation process. In contrast, designers working solo or for a start-up cover a much more comprehensive range of activities and have to cross the disciplinary boundaries of design, adapting to the specific conditions of the new venture.

***Design entrepreneurship without a discipline.*** Nowadays, design practices should move far from linear methodologies ( Bremner & Rodgers, 2013) or prescriptive models. Design has shifted from ‘disciplinary based’ to ’project-based’ ( Heppell, 2006). Design entrepreneurship requires the designer´s ability to combine ideas and methods from different areas of knowledge and keep themselves in a constant learning loop. Feyerabend (2010) describes the designer’s mindset as an ’anything goes’ mindset that is not inhibited by well-confirmed theories or established working practices. Designer entrepreneurs fit within this description. Certain conditions contribute to this flexible mindset such as advancements in prototyping technology, global connectivity, access to funding via crowdfunding platforms and information accessibility ( Valencia & Pearce, 2019), facilitating learning through trial and error. According to Kelly & Kelly (2013), prototyping and testing with the user is one of design thinking's key principles, which can reveal problems sooner and enable learning.

It is worth noting that, in this un-disciplined state of loose methods described by Bremner & Rodgers (2013), designers need a compass that helps them navigate through the uncertainty of product development and venture creation. User centricities satisfy this need in product design; however, there is no evidence that this can be applied to design entrepreneurship.

## Methods

### Methodological approach

This study followed the Constructivist Grounded Theory (CGT) approach proposed by Charmaz (2006). This research strategy requires the researcher to come open-minded but not empty-minded. Charmaz’s version of CGT encourages the researcher to do research beforehand and be flexible in the data generation model, asking off-script questions and bringing spontaneous reflections to the interview. CGT encourages the researcher’s exposure to the available literature and theoretical frameworks, contrary to the conventional Grounded Theory principles of avoiding the literature and conceptual models. CGT uses new insights, emergent questions, and further information to construct not only the method of data generation but also analysing the data simultaneously ( Charmaz, 2006; Charmaz, 2008). Charmaz advocates that the grounded theorist can adapt strategies depending on the demands of the study. This strategy allowed insights to emerge, and its flexibility allowed additions and adaptations such as the use of visual prompts in the inquiry (see below), which also enabled the visual analysis of the information.

The principal investigator, which at the time was studying his PhD, was in charged to collect the data (interviews and activities). A mixed research team was put together in the study - 2 males, 1 female. The postgraduate school at Northumbria University provided workshops and training to the principal investigator to develop the research skills necessary for this study.

### Research method

***Study participants.*** In phase 2, the research team selected individuals with an experience in design or entrepreneurship, principally from the United Kingdom, as a purposive sampling technique ( Robinson, 2014). Three organisations served as facilitators in identifying participants: the research’s host university, an influential design charity and a product design investment fund, all in the UK. The participants consisted of four academic experts in design, seven academic experts in entrepreneurship, 11 designer entrepreneurs, four product-oriented investors, four non-designer entrepreneurs, five head of incubation programmes and one head of a crowdfunding platform.

For phase 3, the research team followed up the approach with designer entrepreneurs from phase 2. These participants covered the following criteria: D-entrepreneur, working in a consumer products start-up (tangible products, non-perishables), with at least one product in the marketplace at the time of the interview. For phase 4, one expert in design, one in entrepreneurship and one in research methods gave their opinions on the findings of the investigation.

The ethics committee of Northumbria University revisited this study to ensure it complied with ethical university policies. The committee granted the ethical approval before the data generation phase was started. The participants in the study gave written informed consent to the research team at the beginning of the study and verbal consent before each interview.

### Structure and data generation methods

The study consisted of four phases. Phase 1 reviewed the available studies in the subject and compiled them in a map of doodles that captured the critical insights of the literature review. Phase 2 consisted of an interview model that explored the relevant areas of the entrepreneurial journey with participants involved in design, entrepreneurship, and innovation. Phase 3 used a semi-structured interview ( Valencia, 2021) and a visual map of possible milestones, targeted specifically to designer-entrepreneurs to help them describe their entrepreneurial journey. A think-aloud activity accompanied this map to help D-entrepreneurs articulate their key learnings on each specific milestone, the sequence, and the journey’s challenges. Phase 4 addressed the validity of the study. These are described below.

***Phase 1: literature review and respondent recruitment.*** The research team conducted a systematic literature review to find the commonalities between the ‘design approach’ and contemporary theories of entrepreneurship. Valencia *et al.* (2018) created a typology of design innovation for consumer product innovation, encompassing relevant theories such as effectuation and causation ( Sarasvathy, 2001), bricolage ( Baker & Nelson, 2005), strategic design ( Calabretta *et al.*, 2016), and design thinking ( Brown, 2008). Based on this typology, the study integrated a set of questions into a semi-structured interview followed by a group of activities to generate data. An initial map of doodles was created to document progress in the investigations.

After the first phase, the doodle map evolved with each interview, becoming not only descriptive but also an analytical tool. Additionally, by creating a short voice-over video of the doodle map, the research team was able to attract study participants by creating an attractive visual aid rather than purely using a study invitation email. The video was distributed on social media platforms such as LinkedIn and from the personal email of the principal investigator. The short videos explained the research context, the relevant theories and gaps found in the literature, and also why the participants’ expertise was needed to fill out the missing gaps.

***Phase 2: interviews.*** For phase 2, an interview model was created based on the findings of the literature review in phase one. The semi-structured interviews served to enable an understanding of the opinions and experiences of individuals related to design, entrepreneurship and innovation in the UK ecosystem. Based on these interviews, the doodle map created in phase 1 evolved with each subsequent interview/encounter; it became a depository of the new insights and findings of the study. The flexibility in the research process opened further inquiries. The iterative approach followed in data generation during phase 2 enhanced the construction of the think-aloud protocol ( Ericsson & Simon, 1980) and milestone map used in data generation during phase 3.

***Phase 3: think-aloud milestone map.*** Using the think-aloud protocol tool developed by Ericsson & Simon (1980), the research team asked participants to describe their entrepreneurial journey using a visual map, to expand on the data generated in phase 2. Phase 3 of this study was conducted at least one month after completion of phase 2, to allow participants to reflect on their entrepreneurial journey. For this phase, the research team created a visual map of 24 entrepreneurial milestones, formed using a combination of the elements of the eight innovation processes from Salerno *et al.* (2015), the lean start-up methodology ( Ries, 2011), the start-up evolution curve ( Jonikas, 2017) and the pre-production milestones of manufacturing products ( Miller, 2016). The study utilised this map to allow D-entrepreneurs to recreate their journey in a think-aloud protocol activity. The participant had to connect the milestones chronologically while verbally describing each milestone’s challenges, decisions, and learning opportunities. Table 1 shows the combined milestones of the entrepreneurial journey, compiled by the authors based on Jonikas (2017); Miller (2016); Salerno *et al.* (2015) and Ries (2011), and used to build the visual map for phase 3 of the data generation.

**Table 1.** The milestones of the entrepreneurial journey visual map (compiled by the authors based on Jonikas, 2017; Miller, 2016; Salerno *et al.*, (2015) and Ries, 2011).

Milestones	Description
Idea generation	Considered as the systematic search for new product ideas ( Law, 2009), yet it can be unsystematic or spontaneous.
User research	The research concentrates on user behaviours, needs, and motivations through observation techniques, task analysis, and other feedback methodologies ( Goodman *et al.*, 2012; Ries, 2011).
Product dev.	It consists of turning a prototype or concept into a workable market offering ( Rouse, 2019). This milestone can extend in time, it is expected that the participant shows the starting and ending point.
Funding	This stage provides financial support to start-ups to finance the project.
Rise capital	This stage refers to the money obtained externally to get the business off the ground and help the daily operations.
Validation	The validation indicates the assessment of the idea, product or the start-up and acceptance from potential customers ( Ries, 2011).
Crowdfunding	In this research, crowdfunding is a way to raise finance from a large number of people, typically using an online platform, where the project is subject to pledges ( Kurani, 2021).
Pivoting	Pivot refers to more substantive iteration ( Ries, 2011). This stage refers to the abrupt change that companies may make to their business model, in response to or in anticipation of a change in the market.
Minimum viable product (MVP)	The MVP allows the start-up to collect feedback and validated learning from customers with the most reduced version of a product ( Ries, 2011).
Mentorship	The mentorship stage is when a mentor influence, guide, or directs the designer-entrepreneur ( Jonikas, 2017).
Diffusion	The diffusion stage refers to the communication process in which the entrepreneurs explain their ideas, information, product and start-up to their community or society ( Salerno *et al.*, (2015))
Wait to develop the market	The entrepreneur decides to stop other areas of the business to develop the existing market rather than looking for a new market ( Salerno *et al.*, (2015)).
Wait to develop the tech.	The entrepreneur decides to stop other areas of the business to develop the technology by systematic use of scientific, technical, economic, and commercial knowledge to meet specific business objectives or requirements ( Salerno *et al.*, (2015)).
Outsource	This stage indicates the practice of subcontracting another company to perform services and create goods that cannot be performed in-house.
Manu-facturing	This stage points out the process of converting materials, components, or parts into the finished product ( Miller, 2016; Salerno *et al.*, (2015)).
Sell	This milestone indicates the exchange of money for the final product. It can be online, in a departmental store or in an independent store.
Distribution	This stage is representative of the milestone of moving the product through a distribution channel to the final customer, customer, or user ( Salerno *et al.*, (2015)).
Intellectual property	This milestone represents the need to protect the creative idea from entrepreneurs ( Jonikas, 2017).
Rapid prototyping	Designers utilized sketches, tangible models, or computer-generated models to configurate a rough-and-ready prototype ( Ries, 2011).
Market research	This milestone refers to the activity of identifying the size of the market, the user´s unmet needs, and potential threats for the company, and market opportunities.
Resources evaluation	This research refers to the resource evaluation milestone to the activity where entrepreneurs evaluate tier resources: materials, human capital, tools, and funds.
Engineering validation test (EVT)	EVT evaluates the assembly of the parts for fit and tests the product for function. The hypothesis of the core engineering functions is tested ( Henning, 2020; Miller, 2016).
Design validation test	The production line is built and tested. The test covers the production lines and whether or not they are able to produce and end unit that meets all the product requirements ( Henning, 2020; Miller, 2016).
Production validation test	At this stage the production line is tested to show how the production process work at scale ( Henning, 2020; Miller, 2016).

*Phase 4: the trustworthiness of the study*

To demonstrate the trustworthiness of the study, the research team followed the recommendations of Charmaz (2006); Moerman (2016); Shenton (2004); Sikolia *et al.* (2013), conducting activities for internal and external validation. To secure a code-recode strategy, the researcher conducted two coding processes, separated in time to allow the ’gestation period’, and then compared the results. This activity was carried out using a small sample of data. The transcripts of the interviews were shown to the participants for their approval. To secure stepwise replication, the researcher asked four external researchers to analyse the same data, noting a slight discrepancy between the data and the codes that emerged from the research team analysis. For the peer examination, the principal investigator actively participated in seminars and presented this work among researchers to receive feedback about the process and findings of the study. Expert external design researchers performed an audit trail on the research and its conclusions; they had access to the raw data, memos, and evidence to track any decision made by the researcher. To comply with the external validation, the researchers created a diagram summarising the insights and the milestone sequence expressed in phase 2 and 3 by each of the participants, and then showed it to the three experts in business, design, and research methodology, respectively, to hear their comments on the investigation and the relevance of the findings.

***Sampling approach in three phases.*** A semi-structured interview in phase 1 helped the research team to gain insight into the entrepreneurial journey of designer-entrepreneurs. The researcher recorded the key insights of the interviews, memos, opinions, and visual feedback in doodles to make sense of the information provided by the participants visually. The visual support enabled the D-entrepreneurs to tell stories and be more descriptive in their answers. This tool was critical to discover deeper insights. Researchers note that the use of maps and diagrams can be used as a form of inquiry and as a cognitive tool to improve memory and processing of information ( Larkin & Simon, 1987; Tversky & Lee, 1998). Visual methods have long been used to generate data in the social sciences ( Warren, 2009).

### Data analysis

CGT recommends the generation of data and its simultaneous analysis before collecting the whole sample, thus enabling conceptualisation of the phenomena ( Charmaz, 2006). In this case, the map of doodles and the visual memos served this purpose. The recorded audio from multiple interviews were 46 hrs long in total. There were periods when the researchers analysed the data collected while other participants joined the study. This iterative, parallel process optimised the time and resources of the researchers, and consequently, the conceptualisation of the phenomena became more robust. This conceptualisation brought new questions and reflections to the interviews, making them more dynamic and reflecting the researchers’ learnings after each interview. The study utilised NVIVO software to analyse the data, (an open source option is Google sheets). This platform facilitates the emergent coding, theoretical coding, data analysis, theoretical development, and presentation of findings ( Hutchisona *et al.*, 2010). With direct quotes from the data, the researchers integrated field notes and diagrams to correlate and strengthen the credibility of the interpretation of the data ( Thomas & Magilvy, 2011; Tuckett, 2005). The coding process followed the recommendation given by Charmaz of using gerunds because they "move beyond concrete statements by focusing on actions rather than themes" ( Charmaz, 2014, 111).

## Results

In CGT, data generation and analysis take place simultaneously. Therefore, in this paper, the ongoing findings are reported as a continuum.

**Figure 1.**
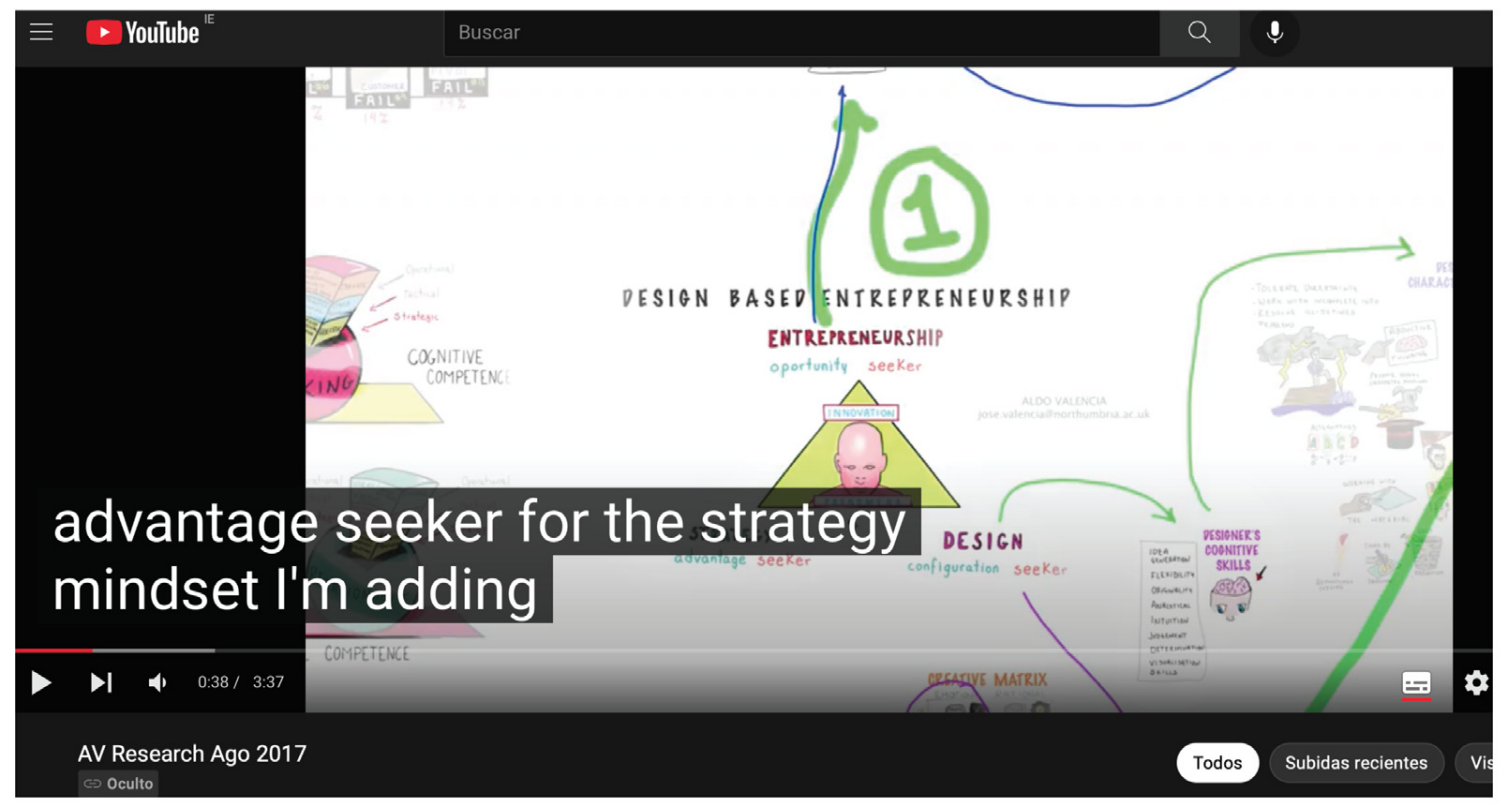
Voice-over video of the doodle map.

In phase 1 and phase 2, the research team summarised the findings in a map of doodles. The principal investigator created this map from the literature review (phase 1), and then it was subjected to changes during and after each interview (phase 2). It was used as a descriptive tool and research prompt, as well as an analytical tool. Figure 1 shows screenshots of the voice-over video created to disseminate the latest findings and to invite participants to the study over social media. This video showed participants the connections between the key concepts and the emergent findings of the inquiry. Participants reported that reviewing the doodles was more appealing than reading written reports, as it let them make sense of the entire scope of the research.

**Figure 2.**
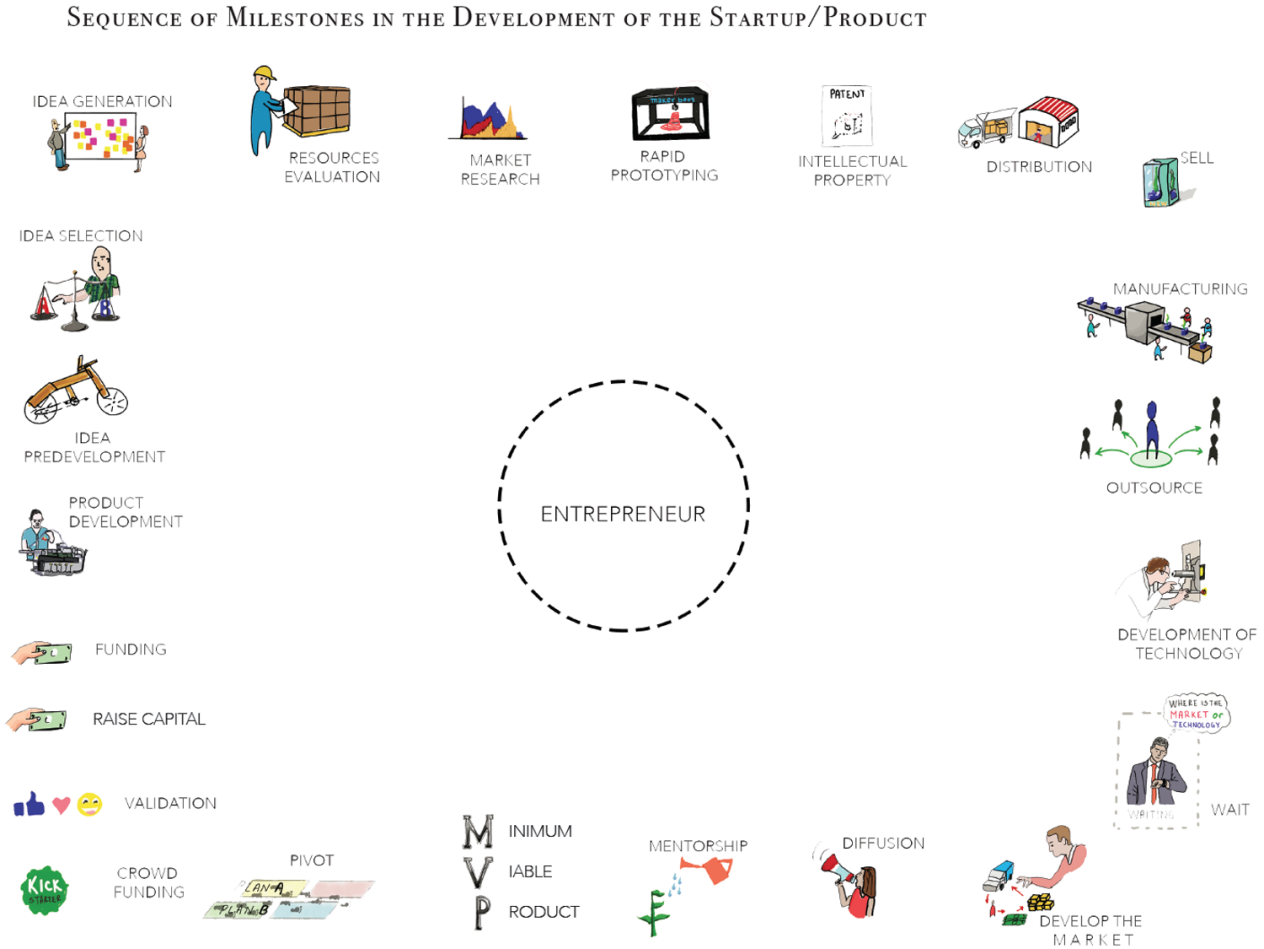
Visual entrepreneurial milestones activity.

Figure 2 shows the visual milestone activity in phase 3. The imagery had to utilise colours, shapes, and simple forms to allow the participant to focus on recalling their process, instead of reading the definition of each.

**Figure 3.**
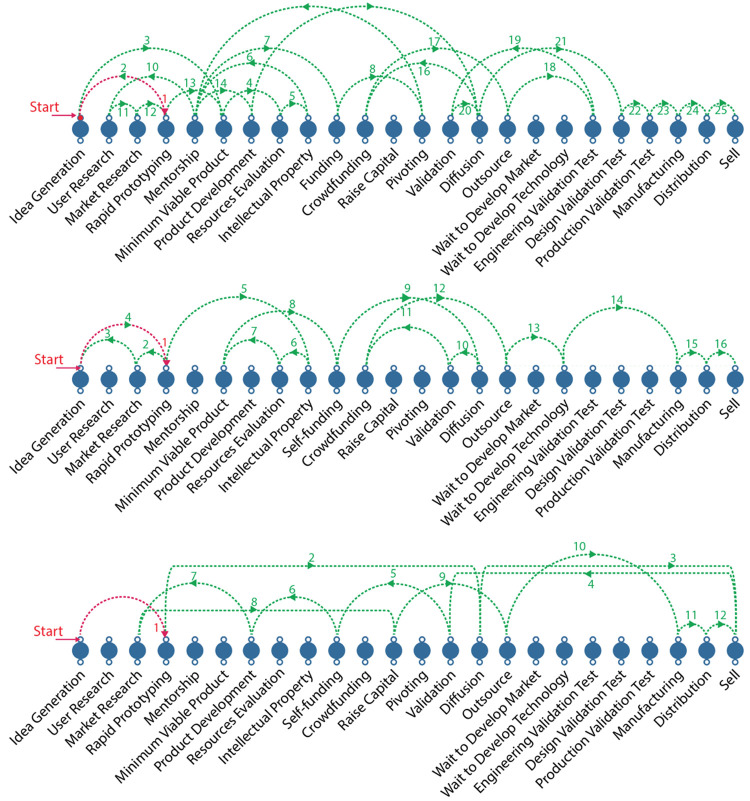
Exploded view of the milestone´s sequence of the three d-entrepreneurs´ journey.

Figure 3 shows examples coming from the D-entrepreneurs in the study. It is worth noticing that each entrepreneurial journey differs from each other. In a subsequent meeting, the results were shown to the participants to collect their impressions and compared the accuracy of the data generated.

There was no consistent milestone sequence among all participants. The only clear pattern was that seven of 11 D-entrepreneurs started their company without following any user-centric method or business plan. One participant asked for feedback for the first time only after one and a half years of product development. These designers were driven by their intuition, their convictions, and their beliefs. The opportunity seemed to be revealed to them while they sketched concepts, explored modern technologies and new materials.

To an extent, they represented the lead user as a source of innovative progress described by Von Hippel (1986) since they had needs ahead of any existent trend and pursued a benefit by obtaining a solution to that need. However, there was a hidden motivation that related to the act of doing, making, and creating. The data obtained suggested something was leading designers towards building personally driven products, regardless of the market, the user insights or if the technology had been proven.

### Codes, categories, and themes

Multiple themes emerged from the data analysis pointing out the subprocesses of the entrepreneurial journey, the challenges, and the lessons learned. However, this paper will focus on the theme ‘authorship’ that resulted to be the most relevant for the study. In Table 2, a breakdown of the authorship theme is presented.

**Table 2.** The authorship categories and gerund codes which emerged from phase 3.

Theme	Category	Gerund Codes
Authorship	Quality, merit and aesthetic obsession	Focusing on details Perfecting over progressing
	Identity	Believing and valuing Telling credible stories Motivating inner self
	Design acumen	Savvy audience criticizing Legitimatizing Empathizing systematically

## Conclusion

### The D-entrepreneur´s authorship theory

The authors posit a new concept, ‘Design Authorship’ (D-authorship), as an intrinsic driver that motivates designers to take the leap into the entrepreneurial journey regardless of user research, marketing study, or any predicted commercial success. Evidence in this study shows that some d-entrepreneurs replace user-centricity with their personal ethos, needs and aspirations as a key driver of the entrepreneurial journey.

A designer-entrepreneur’s authorship is divided into three components: craft, design and artistic. The artistic component of D-authorship portrays the philosophical stand of the entrepreneur. It does not follow any external brief and is mostly based on the D-entrepreneur’s context and personal values. The product/start-up satisfies the designer’s emotional needs, providing meaning and alignment to their values and context. The design element of authorship seeks alignment with brand values, follows an external brief, and pursues social validation. The client/user needs are at the forefront of the priorities. The craft element of authorship concentrates on the designer’s attention to the mastery of execution, the aesthetic response and the merit attained by the skills and taste of the designer. The design flair and good taste reside within this component.

**Figure 4.**
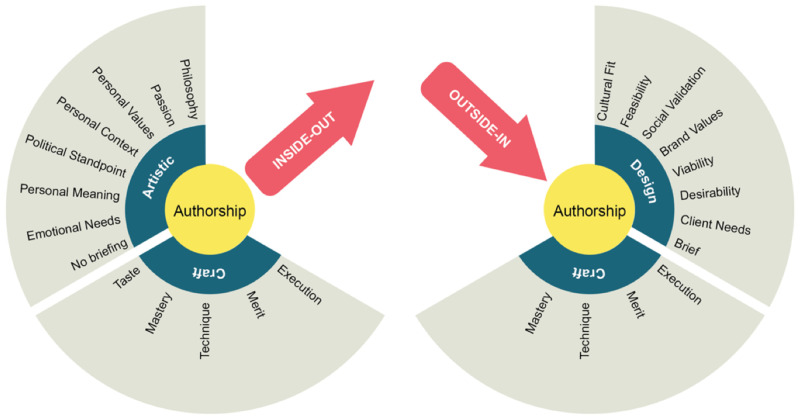
On the left-hand side, the inside-out authorship called the Geppetto Effect; on the right-hand side, the outside-in authorship called the Shoemaker Effect.

***Inside-out authorship (The Geppetto Effect).*** Seven of eleven D-entrepreneurs in this study conceived their products as an extension of who they were, passing on the beliefs and capabilities as designers to the products they created. This phenomenon has been named ‘The Geppetto Effect’ ( Valencia Hernandez & Pearce, 2019). The D-entrepreneurs spent considerable time in expressing perfection, diligence and a need to achieve a sense of authorship through the purpose and characteristics of the product. This ongoing search for perfection slowed the entrepreneurial venture, but it gained authenticity, which later on was needed to appeal to potential users. Shown on the left-hand side of Figure 4, where the D-entrepreneur´s values shape the object and the company, this process is more intimate with the individual ethos. The D-entrepreneurs under this effect took each product decision very carefully. They worked hard to achieve alignment or coherence between the product and their vision and intent. In effect, this was an inside-out process since these designers created items that were meaningful to themselves.

D-entrepreneurs spent more time finding the solutions within themselves, crafting the product up to a point to transfer their identity to the object. This type of authorship represents a mixed blessing, where the designer’s search for perfection and attention to product detailing hindered the start-up’s progress. However, designers with this type of authorship achieved outstanding recognition from their communities. There is an evident coherence between ‘the ethos’ of the product, the start-up and the ‘mastermind’ behind them. It is worth noting that D-entrepreneurs with artistic authorship considered their peers (knowledgeable designers) to be their audience. Multiple contests and prizes, even recognition from international authorities in the design discipline, helped them to build a good reputation even when sales were scarce.

***Outside-in authorship (The Shoemaker Effect).*** The second type of authorship describes when a product results from a systematic process such as design thinking. In this case, D-entrepreneurs play the role of interpreters, collecting information about needs and opinions to form a better understanding of the problem and the potential for future solutions. The researchers called this ‘the designer´s authorship’ as shown on the right-hand side of Figure 4. In this process, the answer comes from the users and the designer´s ability to synthesise abstract information and configure a solution. This is an outside-in process, where the information and validation come from the outside world. This authorship appraises viability, desirability, and feasibility, which accelerate the development process. This study calls this effect as ‘The Shoemaker Effect’ from the Brothers Grimm fairy tale ‘The Elves & the Shoemaker (first published in 1812). In it, elves and a shoemaker secretly collaborate to make shoes that appeal better to customers. Shoemaking is a user-driven activity that builds on a bespoke solution that fits the customers’ needs and desires.

The sense of authorship has not been covered in entrepreneurial studies. Craftspeople, artists, and designers express their thoughts by creating. This study refers to the sense of authorship to the creators´ signature that represents a potential legacy, tradition, or reputation.

### Practical implications

The study of D-authorship can elicit new ways for designers to start a company, without considering user-centric methodologies in the very early stages of the venture. Business incubators can rely on the evidence from this paper to further understand the entrepreneurial journey of highly creative individuals. Further research is needed to understand how successful this approach is in non-D-entrepreneurs. D-authorship can also provide guidance in the way design and business schools approach innovation and entrepreneurship. Business schools could learn more about entrepreneurial paths that aren't yet understood by design schools.

## Data availability

### Underlying data

Due to the commercial and intellectual sensitivity of the data handled in this study, all the interviews, transcripts and memos have been stored on the GETM3 data repository at Northumbria University secure servers, as required by our confidential obligations with the EU Commission.

Any further queries or to request access to the data please contact Dr. Aldo Valencia at aldo.valencia@mu.ie. The data access request will be assessed by the GETM3 project committee to comply with our confidentiality obligations with the EU project guidelines. 

### Extended data

Figshare: Semi-structured Interview Study DeEntr.docx. https://doi.org/10.6084/m9.figshare.16775719.v1 ( Valencia, 2021)

This project contains the following extended data:

- Semi-structured Interview Study DeEntr.docx (semi-structured interview model)

Data are available under the terms of the Creative Commons Attribution 4.0 International license (CC-BY 4.0).
