# Supplementary material for: Design Authorship: an intrinsic driver of designer-entrepreneurs
Source: Open Res Eur. 2023 Dec 20;1:133. Originally published 2021 Nov 2. [Version 2] doi: 10.12688/openreseurope.14148.2 (PMC10839857; doi:10.12688/openreseurope.14148.2)
Supplement: Peer review history

# Reviewer report: Reviewer response for version 2

**DOI:** 10.21956/openreseurope.18172.r37526

**Published:** 2024-03-05

**Authors:** Michael G. Goldsby

**Affiliations:** 1 Ball State University, Muncie, Indiana, USA

The article is very well-written and presents a good topic to explore. The inside-out and outside-in framework is very interesting and worth examining. I think this is a very important question to address in design and related entrepreneurship research. A typology based on design within entrepreneurship is a good contribution on this topic. My suggestions to improve this paper: In the Introduction, you are missing citing Design-Centered Entrepreneurship by Basadur, Goldsby, and Mathews (2022). There is much on the thinking around this blend in that book. In the Introduction, more clarification on why the word "authorship" was used. I'm not clear on that from either a design or entrepreneurship angle. Can you give an example of what Design Authorship looks like. Can the authors provide an example of inside-out and outside-in design to better explain how the idea works. Any research literature on authorship to ground the concept more? Later in the paper, Phase 2: I think more detail on what asked in the interviews would help the reader.

If applicable, is the statistical analysis and its interpretation appropriate?

Yes

Is the study design appropriate and is the work technically sound?

Yes

Is the work clearly and accurately presented and does it engage with the current literature?

Yes

Are the conclusions drawn adequately supported by the results?

Yes

Are sufficient details of methods and analysis provided to allow replication by others?

Not applicable

Are all the source data and materials underlying the results available?

Yes

Reviewer Expertise:

Entrepreneurship

I confirm that I have read this submission and believe that I have an appropriate level of expertise to confirm that it is of an acceptable scientific standard, however I have significant reservations, as outlined above.

# Reviewer report: Reviewer response for version 2

**DOI:** 10.21956/openreseurope.18172.r37535

**Published:** 2024-01-29

**Authors:** P.J. White

**Affiliations:** 1 McMaster University (Ringgold ID: 3710), Hamilton, Ontario, Canada; 2 designCORE, Humanities, South East Technological University, Carlow, Ireland

This is an interesting contribution to the area of Design entrepreneurship, and will be of interest to Design and entrepreneurship researchers.  Having read through the paper and comments by reviewers I'm satisfied that the previous amendments are complete and improve the paper. Some slight amendments are required (detailed below) and I would strongly suggest a full final proofread to ensure all spellings, grammar and syntax are correct.

 Suggested Amendments:

 1. In the conclusion I feel the following passage is a main finding of the study and is of importance for future researchers, particularly design researchers:

   “Evidence in this study shows that some d-entrepreneurs replace user-centricity with their personal ethos, needs and aspirations as a key driver of the entrepreneurial journey”

 However, it gets lost in the current formatting/paragraph. For the ease of the reader, I suggest drawing the reader’s attention to it more, either by highlighting it, calling it a main finding etc.

 2. Change of spelling/ referencing

 Paragraph 2 Change spelling Noumerous to Numerous

 Paragraph 13 Helmholtz (1826) needs to be listed in the references?

 3. Please conduct final full proof read for further errors.

 In conclusion, well done, looking forward to seeing how this research develops.

 P.J.

If applicable, is the statistical analysis and its interpretation appropriate?

Not applicable

Is the study design appropriate and is the work technically sound?

Yes

Is the work clearly and accurately presented and does it engage with the current literature?

Yes

Are the conclusions drawn adequately supported by the results?

Yes

Are sufficient details of methods and analysis provided to allow replication by others?

Yes

Are all the source data and materials underlying the results available?

No source data required

Reviewer Expertise:

Design, Product Design, Design for Ageing, Design Ethnography, Co-Design, Interdisciplinary Research

I confirm that I have read this submission and believe that I have an appropriate level of expertise to confirm that it is of an acceptable scientific standard.

# Reviewer report: Reviewer response for version 2

**DOI:** 10.21956/openreseurope.18172.r36929

**Published:** 2024-01-08

**Authors:** Thomas Cooney

**Affiliations:** 1 College of Business, Technological University Dublin (TU Dublin), Dublin, Ireland

I have read the revised article and I am satisfied with the changes that have been made.

If applicable, is the statistical analysis and its interpretation appropriate?

Yes

Is the study design appropriate and is the work technically sound?

Yes

Is the work clearly and accurately presented and does it engage with the current literature?

Partly

Are the conclusions drawn adequately supported by the results?

Partly

Are sufficient details of methods and analysis provided to allow replication by others?

Yes

Are all the source data and materials underlying the results available?

Yes

Reviewer Expertise:

Entrepreneurship

I confirm that I have read this submission and believe that I have an appropriate level of expertise to confirm that it is of an acceptable scientific standard.

# Reviewer report: Reviewer response for version 1

**DOI:** 10.21956/openreseurope.15253.r28046

**Published:** 2021-12-07

**Authors:** Thomas Cooney

**Affiliations:** 1 College of Business, Technological University Dublin (TU Dublin), Dublin, Ireland

This article explores the entrepreneurial journey of product designers to understand the gap between invention and commercialization. Following interviews with eleven design entrepreneurs, the authors develop a new concept called ‘Design Authorship’ (D-authorship) which can either be Inside-Out (design-led) or Outside-In (user-led). 

 The premise of this paper is really interesting as the gap between invention and commercialization remains a constant conundrum. However, the paper needs to be much more tightly written and more attention needs to be given to the value of the findings.

 The following points should assist the authors in the future development of the article:

1. This is a scientific paper and the language used should reflect it. Expressions such as 'go the extra mile',  'tap into', 'that concern with what will exist' and 'right up to' should be be rewritten to a more formal language.
2.  A useful technique when writing an article is to keep asking 'So What?' Having written a paragraph or section, the writer / reader should ask 'so what' to determine what value arises from the information provided. At the end of the literature review, I asked myself 'so what have I learned from the review?' and the answer was unclear. The article did not draw together the main points that had been learned from the review of the literature and how the review advanced the development of the article. For example, would it have been helpful to include a doodle map as was mentioned later in the article?
3. The lean start-up approach by Ries (2011) is very similar to the argument being made in this article about an 'outside-in' approach to design authorship. Therefore, it should have been given specific attention in the literature review and later the authors should have explained the differences between the two approaches.
4. The Research Method section begins with the words 'In Phase 2', but we have not been told previously what is Phase 1. Additionally, we are taken through the four phases in Research Method, only for it to be repeated in more detail in 'Structure and Data Generation Methods'. Furthermore, the article is clearly about the feedback from the eleven design entrepreneurs, so why is there is a need to discuss all the other participants / interviews since none of the information generated from these was actually used.
5. The Conclusion was quite weak and failed to highlight the real value that this work adds to existing academic literature and the implications that it has for practice. There was so much to be written about the practical implications, but only two sentences were given before moving on to the need for future research.

This research and article has the potential to make a really interesting contribution to discussions on the gap between invention and commercialization, but currently it undersells itself by not drawing out more strongly the findings from the research and their implications for practice and theory.

If applicable, is the statistical analysis and its interpretation appropriate?

Yes

Is the study design appropriate and is the work technically sound?

Yes

Is the work clearly and accurately presented and does it engage with the current literature?

Partly

Are the conclusions drawn adequately supported by the results?

Partly

Are sufficient details of methods and analysis provided to allow replication by others?

Yes

Are all the source data and materials underlying the results available?

Yes

Reviewer Expertise:

Entrepreneurship

I confirm that I have read this submission and believe that I have an appropriate level of expertise to confirm that it is of an acceptable scientific standard, however I have significant reservations, as outlined above.

## References

[1] : The Lean Startup: How Today's Entrepreneurs Use Continuous Innovation to Create Radically Successful Businesses. *Crown Business* .2011;

# Response

**Published:** 2023-11-03

**Authors:** Aldo Valencia

**Affiliations:** Business and Law, Northumbria University, Newcastle upon Tyne, England, UK

Dear Thomas Cooney, College of Business, Technological University Dublin (TU Dublin), Dublin, Ireland

 I would like to express my sincere gratitude for the insightful and meticulous review of this paper. Your detailed and constructive feedback has been immensely valuable in refining and strengthening the quality of this manuscript. I appreciate the time and effort you have dedicated to carefully evaluating the content. Please find below the answers of all the comments. Comments and their responses ordered by section of the paper.

**Literature Review** - Comments/Actions:

**- "At the end of the literature review, I asked myself 'so what have I learned from the review?' and the answer was unclear. The article did not draw together the main points that had been learned from the review of the literature and how the review advanced the development of the article. Would it have been helpful to include a doodle map as was mentioned later in the article?"**

Response: The conclusion of the literature review section has been revised to offer a clearer summary of the main points derived from the review. Additionally, a brief section has been added to explicitly emphasize the contributions of the literature review to the overall development of the article. Moreover, an incorporation of a reference to the doodle map, described in the Methods and Research Method section, serves to illustrate how it facilitates the visualization of the conceptual framework of the article

**- "The lean start-up approach by Ries (2011) is very similar to the argument being made in this article about an 'outside-in' approach to design authorship. Therefore, it should have been given specific attention in the literature review and later the authors should have explained the differences between the two approaches."**

Response: A specific section has been included in the literature review that discusses the similarities and differences between the Lean Start-up approach by Ries (2011) and the 'outside-in' approach to design authorship proposed in our article

 - "First, contextualize the concept with regards to existing concepts. For example, which concepts are identified as being related to it? This, then, would help the reader better evaluate the novelty of the concept. A better grounding of the concept would also help in creating a more coherent body of knowledge (instead of separate contributions here and there)."

 - "The introduction could more clearly state the paper focuses on design authorship – after having finished the manuscript, I realized the paragraph “In this paper, we question…” (p. 3) refers to the concept, but it should be made even more explicit. Right now, the introduction seems rather convoluted with discussions on design thinking, user-centricity, entrepreneurs, and design entrepreneurs (same is reflected in keywords that, after revising the manuscript, could be changed to better reflect the manuscript)." 

Response: The section on design authorship in the literature review has been expanded to provide a more thorough contextualization of the concept.

 Related concepts and theories, such as design thinking, user-centricity, and entrepreneurship, have been identified, and the way in which design authorship fits within this conceptual landscape has been discussed. 

 This contextualization enhances the reader's understanding of the novelty and relevance of the concept and contributes to a more coherent body of knowledge.

 - "Literature review, then, seems to focus on comparing entrepreneurs and design entrepreneurs (one idea could be to have a table comparing them if this path is what the authors wish to embark on), but this seems to be slightly misaligned with the introduction. In other words, a) what do we know about design entrepreneurs and b) what we need to know more about?" 

Response: The need for clarity regarding the focus of the literature review has been pointed out, and in response, the literature review section has been restructured to provide a more organized and coherent discussion. A table has been included that compares entrepreneurs and design entrepreneurs to highlight their similarities and differences, addressing what is known about design entrepreneurs and emphasizing the aspects that require further exploration. This revision ensures better alignment with the introduction and addresses the concerns raised by the reviewer about the article's direction.  

**Research Methods** - Comments/Actions:

 "...Method, only for it to be repeated in more detail in 'Structure and Data Generation Methods'. Furthermore, the article is clearly about the feedback from the eleven design entrepreneurs, so why is there a need to discuss all the other participants / interviews since none of the information generated from these was actually used."

Response: The Research Method section provide a more concise and focused overview of the research methodology. Constructivist grounded theory requires the inclusion of different voices related to the subject of study.

**- "The Research Method section begins with the words 'In Phase 2', but we have not been told previously what is Phase 1. Additionally, we are taken through the four phases in Research."**

Response: The research method section has been updated to provide a clear introduction to Phase 1 and a brief overview of the four research phases at the beginning of the section. This provides readers with a better understanding of the research process.  

 - "Visuals, for instance, could be made smaller and some of them could perhaps even be removed (e.g., figures 1 and 2)."   

Response: The former figure 1 has been removed. The removal of this image does not compromise the clarity of the section.

 - "The method section could benefit from expanding it a bit whilst mostly focusing on making it more condensed (I know this is paradoxical, but I will suggest next how this could be achieved). For example, Table 1 could be moved to the Appendix, while Phase 1 could potentially be removed since that should be covered in the literature review section."  

Response: Moving Table 1 to the Appendix is a reasonable suggestion to streamline the main text. However, it compromises the clarity of this section. The reader needs to see the concepts included in the milestones od the entrepreneurial journey included in the visual activity for data collection.  

 - "If the paper deals with multimodal data (as it seems to be given that both visual and verbal data was collected), data analysis could be more robust in showing how multimodal data was analyzed."   

Response: Constructivist Grounded Theory allows the analysis of multimodal data, integrating textual, visual, and auditory information to conduct the phase analysis. The activity milestones show the sequence of events that the entrepreneurs followed. So we treat this visual aids as prompts for the main interview.    

      

**Conclusion** - Comments/Actions:

**- "The Conclusion was quite weak and failed to highlight the real value that this work adds to existing academic literature and the implications that it has for practice. There was so much to be written about the practical implications, but only two sentences were given before moving on to the need for future research."**

Response: We strengthened the conclusion by highlighting the unique contributions of the research to existing academic literature and emphasizing the practical implications of the findings. We expanded on the practical implications, providing a more detailed discussion of how the concept of D-authorship can be applied in practice by designer-entrepreneurs.

 - "...the three components (craft, design, and artistic) are promising, they also require a bit more flesh around the bones. See Suddaby (2010) for potentially useful content on achieving clarity here. More specifically, each of the components should be grounded in extant research and at the same time, they should be complementary instead of overlapping.

Response: These components were re-named to make them consistent (all nouns). The suggested reference was revisited to give clarity to the conclusion section. We precise distinctions between the different elements of the theory. The new concepts were compared to extant literature to look for coherence and semantic relationships.

 - "I would definitely spend more time on developing the design authorship concept as well as devote more space to practical implications. One way this could be achieved might be to condense the methodology section."

Response: This concept has been fleshed out in the conclusion section. Also the practical implication section has been expanded.

 "...design authorship: the concept should be better grounded with regards to extant research (i.e. are there any similar concepts?), the three components could be revised/refined so that it is easier to separate them from each other, and it should be made more explicit whether the concept is binary or spatio-temporally more fluid."

Response: This comment has been addressed in the conclusion section. We included this extract in the first paragraph of the conclusion section. A designer-entrepreneur’s authorship is divided into three components that are complementary and not mutually exclusive.    

          

**Minor corrections** - Comments/Actions:

**- "This research and article has the potential to make a really interesting contribution to discussions on the gap between invention and commercialization, but currently it undersells itself by not drawing out more strongly the findings from the research and their implications for practice and theory"**

Response: okay.

 -" This is a scientific paper and the language used should reflect it. Expressions such as 'go the extra mile', 'tap into', 'that concern with what will exist' and 'right up to' should be rewritten to a more formal language."

Response: Sorted, a proof reader revisit the paper.

 - "What role does materiality play in design authorship? Going back to Schön’s notion of backtalk, how does the material that the designer is working on influence the design authorship? Building on this, the design authorship concept could also benefit from considering the temporal dimension."

Response: Page 22, last paragraph.

 - "Questioning user-centricity is definitely a promising avenue, and then this discussion could be linked in the implications section back to entrepreneurs more broadly."  

Response: Page 23, first paragraph.    

**Minor corrections** - Comments/Actions:

 - "p. 4, “Design entrepreneurs fit within this description” <- how exactly? This could be expanded a bit more." 

Response: Eliminated.

 - "p. 4, “was in charged” <- d should be removed"

Response: Changed.

 - "p. 4, not sure the section on “Design models” is necessary." 

Response: This section has been included to show the reader the connection between the stablished design models and the entrepreneurial milestones.

 - "p. 4, “mixed research team” <- not sure this is necessary if “mixed” in this context only means gender. Instead, maybe it would make sense to describe the authors’ positionality a bit more." 

Response: Changed. Explained in page 9 third paragraph.

 - "Since the study utilizes visuals and verbal accounts, it might be worthwhile to look into multimodal research (e.g. Höllerer et al., 2018; Kress & van Leeuwen, 2020)" 

Response: Constructivist Grounded Theory is a form of Multimodal research. Its emphasis is prioritising the integration of various data sources and data types.

 - "Notions of ethos could be strengthened by drawing on Fayard I.’s (2017) work." 

Response: We include this in Page 20

 - "Would love to see the design authorship concept visualized somehow to increase its chances of getting cited by future research."

Response: There is a video abstract of the D-Authorship https://youtu.be/F3sOyEqp3BI

 - "Think it would make sense to have a more nuanced discussion of designers and design authorship – graphic designers are not the same as fashion designers, and at the same time not every designer is trained to be an artistic designer (thinking of Bruno Munari here)."   

Response: In this research project, this is outside of the scope given the product based designer entrepreneurs.

 - "Flagging it here once more just in case, but how many of the informants were solo entrepreneurs and how many were part of a team?"

Response: All of our designer entrepreneurs had support and collaborators. But in the end they were the ones going through the whole process.

 - "Practical implications could be expanded as there are some very interesting points here! Garbuio *et al*.’s (2018) study on design cognition and entrepreneurship should be useful here." Response: We expanded the practical implications following the previous recommendations.  

 Thank you once again for such a meticulous review 

 Best Regards,

 Dr Aldo Valencia

 Newcastle Business School, Northumbria University, UK.

# Reviewer report: Reviewer response for version 1

**DOI:** 10.21956/openreseurope.15253.r27980

**Published:** 2021-11-16

**Authors:** Miikka Lehtonen

**Affiliations:** 1 Rikkyo University College of Business, Tokyo, Japan

Dear Authors,

 It was a pleasure reading this manuscript titled ”Design Authorship: an intrinsic driver of designer-entrepreneurs”. As is mentioned at the end of the manuscript, this is a topic that warrants further inquiry, and I could not agree more. As such, I hope this review statement helps in further developing this manuscript so it could attract future research even more.

 Before moving on to the actual review statement, sharing a few words about the perspective from where it is coming. In essence, I obtained my PhD from a business in school in 2014, and ever since I have been working mostly in business and design schools. Teaching and researching strategic design / design management as well as writing about design-driven pedagogies. Just to give you a brief idea on what is my perspective to the paper.

 To begin with, the concept of design authorship is both interesting and relevant. To make it even stronger, though, two things might be useful. First, contextualize the concept with regards to existing concepts. For example, which concepts are identified as being related to it? This, then, would help the reader better evaluate the novelty of the concept. In addition, a better grounding of the concept would also help in creating a more coherent body of knowledge (instead of separate contributions here and there). Second, while the three components (craft, design, and artistic) are promising, they also require a bit more flesh around the bones. See Suddaby (2010) for potentially useful content on achieving clarity here. More specifically, each of the components should be grounded in extant research and at the same time they should be complementary instead of overlapping. For instance, Bell *et al.* (2019) have written about craft from management studies’ perspective, and at the same time one could also argue that design can be artistic and contain craft as an integral element. Point being, since the three components are so closely interrelated, why not collapse them into one component? To be clear, I do see value in this concept, just highlighting here that the design authorship concept requires more discussion and a deeper connection to extant research, lest it becomes convoluted and slightly tautological.

 At the same time, and as a minor comment, what role does materiality play in design authorship? Going back to Schön’s notion of backtalk, how does the material that the designer is working on influence the design authorship? Building on this, the design authorship concept could also benefit from considering the temporal dimension. Is design authorship a binary concept? Or does it fluctuate over time?

 To summarize my comments regarding design authorship: the concept should be better grounded with regards to extant research (i.e. are there any similar concepts?), the three components could be revised / refined so that it is easier to separate them from each other, and it should be made more explicit whether the concept is binary or spatio-temporally more fluid.

 Looking at the comments above and below, I would definitely spend more time on developing the design authorship concept as well as devote more space to practical implications. One way this could be achieved might be to condense the methodology section. Visuals, for instance, could be made smaller and some of them could perhaps even be removed (e.g., figures 1 and 2) – as a visual researcher, I am all up for making manuscripts more visual, but at the same time they should somehow move the main argument(s) forward. From this perspective, I am not convinced figures 1 and 2 carry much weight (not saying they are worthless, just not convinced they are needed here).

 At the same time, the method section could benefit from expanding it a bit whilst mostly focusing on making it more condensed (I know this is paradoxical, but I will suggest next how this could be achieved). For example, Table 1 could be moved to the Appendix, while Phase 1 could potentially be removed since that should be covered in the literature review section. At the same time, and echoing one of the minor comments below, if the paper deals with multimodal data (as it seems to be given that both visual and verbal data was collected), data analysis could be more robust in showing how multimodal data was analyzed. In other words, it should be made more explicit how theoretical insights were derived from the data (instead of just telling about the analysis process, it would be great to show this to the reader).

 In terms of positioning the paper, the introduction could more clearly state the paper focuses on design authorship – after having finished the manuscript, I realized the paragraph “In this paper, we question…” (p. 3) refers to the concept, but it should be made even more explicit. Right now, the introduction seems rather convoluted with discussions on design thinking, user-centricity, entrepreneurs, and design entrepreneurs (same is reflect in keywords that, after revising the manuscript, could be changed to better reflect the manuscript). Literature review, then, seems to focus on comparing entrepreneurs and design entrepreneurs (one idea could be to have a table comparing them, if this path is what the authors wish to embark on), but this seems to be slightly misaligned with the introduction. In other words, a) what do we know about design entrepreneurs and b) what we need to know more about? Questioning user-centricity is definitely a promising avenue, and then this discussion could be linked in the implications section back to entrepreneurs more broadly. In essence, it feels as if the beginning of the manuscript is trying to promise a bit too much, but there is no need for that. This is a promising paper that holds great potential on its own!

 All in all, this is an interesting study that will most certainly contribute to our current understanding of design entrepreneurship. Many thanks for inviting me on this journey, and hope this review statement helps in further developing the manuscript!

 Minor comments:

- p. 4, “Design entrepreneurs fit within this description” <- how exactly? This could be expanded a bit more.
- p. 4, “was in charged” <- d should be removed.
- p. 4, not sure the section on “Design models” is necessary.
- p. 4, “mixed research team” <- not sure this is necessary if “mixed” in this context only means gender. Instead, maybe it would make sense to describe the authors’ positionality a bit more.
- Since the study utilizes visuals and verbal accounts, it might be worthwhile to look into multimodal research (e.g. Höllerer et al., 2018; Kress & van Leeuwen, 2020)
- Notions of ethos could be strengthened by drawing on Fayard I.’s (2017) work.
- Would love to see the design authorship concept visualized somehow to increase its chances of getting cited by future research.
- Think it would make sense to have a more nuanced discussion of designers and design authorship – graphic designers are not the same as fashion designers, and at the same time not every designer is trained to be an artistic designer (thinking of Bruno Munari here).
- Flagging it here once more just in case, but how many of the informants were solo entrepreneurs and how many were part of a team?
- Building on the above, perhaps there could be a 2x2 matrix, for example, that categorizes design authorship across different categories (e.g., would it make sense to say a designer working in a design agency might manifest “weak” design authorship while a designer working in their own studio is a textbook example of “strong” design authorship? This is just a rough example, in reality the situation is more nuanced).
- Practical implications could be expanded as there are some very interesting points here! Garbuio *et al*.’s (2018) study on design cognition and entrepreneurship should be useful here.

References:

 Bell, E. *et al*. (2019). *The Organization of Craft Work*. Routledge.

 Fayard, A-L. *et al*. (2017). How Nascent Occupations Construct a Mandate: The Case of Service Designers’ Ethos. *Administrative Science Quarterly*, 62(2), 270-303.

 Garbuio, M. *et al*. (2018). Demystifying the Genius of Entrepreneurship: How Design Cognition Can Help Create the Next Generation of Entrepreneurs. *Academy of Management Learning & Education*, 17(1), 41-61.

 Höllerer, M. A. *et al*. (2018). ‘A picture is worth a thousand words’: Multimodal sensemaking of the global financial crisis. *Organization Studies*, 39, 617-644.

 Kress, G. R. & van Leeuwen, T. (2020). *Reading images: The grammar of visual design*. Routledge.

 Suddaby, R. (2010). Editor's Comments: Construct Clarity in Theories of Management and Organization. *Academy of Management Review*, 35(3), 346-357.

If applicable, is the statistical analysis and its interpretation appropriate?

No

Is the study design appropriate and is the work technically sound?

Yes

Is the work clearly and accurately presented and does it engage with the current literature?

Partly

Are the conclusions drawn adequately supported by the results?

Partly

Are sufficient details of methods and analysis provided to allow replication by others?

Partly

Are all the source data and materials underlying the results available?

Partly

Reviewer Expertise:

Design management, creative industries, design education

I confirm that I have read this submission and believe that I have an appropriate level of expertise to confirm that it is of an acceptable scientific standard, however I have significant reservations, as outlined above.

## References

[1] : The Organization of Craft Work: Identities, Meanings, and Materiality. *Routledge* .2019;

[2] : How Nascent Occupations Construct a Mandate: The Case of Service Designers’ Ethos. *Administrative Science Quarterly* .2017;62(2) : 10.1177/0001839216665805 270-303 10.1177/0001839216665805

[3] : Demystifying the Genius of Entrepreneurship: How Design Cognition Can Help Create the Next Generation of Entrepreneurs. *Academy of Management Learning & Education* .2018;17(1) : 10.5465/amle.2016.0040 41-61 10.5465/amle.2016.0040

[4] : ‘A Picture is Worth a Thousand Words’: Multimodal Sensemaking of the Global Financial Crisis. *Organization Studies* .2018;39(5-6) : 10.1177/0170840618765019 617-644 10.1177/0170840618765019

[5] : Reading images: The grammar of visual design. *Routledge* .2020;

[6] Editor's Comments: Construct Clarity in Theories of Management and Organization. *Academy of Management Review* .2010;35(3) : 10.5465/amr.35.3.zok346 346-357 10.5465/amr.35.3.zok346

# Response

**Published:** 2023-11-03

**Authors:** Aldo Valencia

**Affiliations:** Business and Law, Northumbria University, Newcastle upon Tyne, England, UK

Dear Dr Miikka Lehtonen,  Rikkyo University College of Business, Tokyo, Japan.

 I would like to express my sincere gratitude for the insightful and meticulous review of this paper. Your detailed and constructive feedback has been immensely valuable in refining and strengthening the quality of this manuscript. I appreciate the time and effort you have dedicated to carefully evaluating the content. Please find below the answers of all the comments. Comments and their responses ordered by section of the paper.

**Literature Review** - Comments/Actions:

 - "At the end of the literature review, I asked myself 'so what have I learned from the review?' and the answer was unclear. The article did not draw together the main points that had been learned from the review of the literature and how the review advanced the development of the article. Would it have been helpful to include a doodle map as was mentioned later in the article?"  

Response: The conclusion of the literature review section has been revised to offer a clearer summary of the main points derived from the review. Additionally, a brief section has been added to explicitly emphasize the contributions of the literature review to the overall development of the article. Moreover, an incorporation of a reference to the doodle map, described in the Methods and Research Method section, serves to illustrate how it facilitates the visualization of the conceptual framework of the article

 "The lean start-up approach by Ries (2011) is very similar to the argument being made in this article about an 'outside-in' approach to design authorship. Therefore, it should have been given specific attention in the literature review and later the authors should have explained the differences between the two approaches."  

Response: A specific section has been included in the literature review that discusses the similarities and differences between the Lean Start-up approach by Ries (2011) and the 'outside-in' approach to design authorship proposed in our article

 - "First, contextualize the concept with regards to existing concepts. For example, which concepts are identified as being related to it? This, then, would help the reader better evaluate the novelty of the concept. A better grounding of the concept would also help in creating a more coherent body of knowledge (instead of separate contributions here and there)."

 - "The introduction could more clearly state the paper focuses on design authorship – after having finished the manuscript, I realized the paragraph “In this paper, we question…” (p. 3) refers to the concept, but it should be made even more explicit. Right now, the introduction seems rather convoluted with discussions on design thinking, user-centricity, entrepreneurs, and design entrepreneurs (same is reflected in keywords that, after revising the manuscript, could be changed to better reflect the manuscript)."  

Response: The section on design authorship in the literature review has been expanded to provide a more thorough contextualization of the concept.   Related concepts and theories, such as design thinking, user-centricity, and entrepreneurship, have been identified, and the way in which design authorship fits within this conceptual landscape has been discussed.   This contextualization enhances the reader's understanding of the novelty and relevance of the concept and contributes to a more coherent body of knowledge.

 - "Literature review, then, seems to focus on comparing entrepreneurs and design entrepreneurs (one idea could be to have a table comparing them if this path is what the authors wish to embark on), but this seems to be slightly misaligned with the introduction. In other words, a) what do we know about design entrepreneurs and b) what we need to know more about?" 

Response: The need for clarity regarding the focus of the literature review has been pointed out, and in response, the literature review section has been restructured to provide a more organized and coherent discussion. A table has been included that compares entrepreneurs and design entrepreneurs to highlight their similarities and differences, addressing what is known about design entrepreneurs and emphasizing the aspects that require further exploration. This revision ensures better alignment with the introduction and addresses the concerns raised by the reviewer about the article's direction.  

**Research Methods** - Comments/Actions:

 "...Method, only for it to be repeated in more detail in 'Structure and Data Generation Methods'. Furthermore, the article is clearly about the feedback from the eleven design entrepreneurs, so why is there a need to discuss all the other participants / interviews since none of the information generated from these was actually used.

Response: The Research Method section provides a more concise and focused overview of the research methodology. Constructivist grounded theory requires the inclusion of different voices related to the subject of study.

 - "The Research Method section begins with the words 'In Phase 2', but we have not been told previously what is Phase 1. Additionally, we are taken through the four phases in Research..."  

Response: The research method section has been updated to provide a clear introduction to Phase 1 and a brief overview of the four research phases at the beginning of the section. This provides readers with a better understanding of the research process.  

 - Visuals, for instance, could be made smaller and some of them could perhaps even be removed (e.g., figures 1 and 2).    

Response: The former figure 1 has been removed. The removal of this image does not compromise the clarity of the section.

 - "The method section could benefit from expanding it a bit whilst mostly focusing on making it more condensed (I know this is paradoxical, but I will suggest next how this could be achieved). For example, Table 1 could be moved to the Appendix, while Phase 1 could potentially be removed since that should be covered in the literature review section."  

Response: Moving Table 1 to the Appendix is a reasonable suggestion to streamline the main text. However, it compromises the clarity of this section. The reader needs to see the concepts included in the milestones od the entrepreneurial journey included in the visual activity for data collection.  

 - "If the paper deals with multimodal data (as it seems to be given that both visual and verbal data was collected), data analysis could be more robust in showing how multimodal data was analyzed."    

Response: Constructivist Grounded Theory allows the analysis of multimodal data, integrating textual, visual, and auditory information to conduct the phase analysis. The activity milestones show the sequence of events that the entrepreneurs followed. So we treat this visual aids as prompts for the main interview.    

      

**Conclusion** - Comments/Actions:

 - "The Conclusion was quite weak and failed to highlight the real value that this work adds to existing academic literature and the implications that it has for practice. There was so much to be written about the practical implications, but only two sentences were given before moving on to the need for future research".

Response: We strengthened the conclusion by highlighting the unique contributions of the research to existing academic literature and emphasizing the practical implications of the findings. We expanded on the practical implications, providing a more detailed discussion of how the concept of D-authorship can be applied in practice by designer-entrepreneurs.

 - "...the three components (craft, design, and artistic) are promising, they also require a bit more flesh around the bones. See Suddaby (2010) for potentially useful content on achieving clarity here. More specifically, each of the components should be grounded in extant research and at the same time, they should be complementary instead of overlapping."

Response: These components were re-named to make them consistent (all nouns). The suggested reference was revisited to give clarity to the conclusion section. We precise distinctions between the different elements of the theory. The new concepts were compared to extant literature to look for coherence and semantic relationships.

 - "...I would definitely spend more time on developing the design authorship concept as well as devote more space to practical implications. One way this could be achieved might be to condense the methodology section."

Response: This concept has been fleshed out in the conclusion section. Also the practical implication section has been expanded.

 - "...design authorship: the concept should be better grounded with regards to extant research (i.e. are there any similar concepts?), the three components could be revised/refined so that it is easier to separate them from each other, and it should be made more explicit whether the concept is binary or spatio-temporally more fluid."

Response: This comment has been addressed in the conclusion section. We included this extract in the first paragraph of the conclusion section. A designer-entrepreneur’s authorship is divided into three components that are complementary and not mutually exclusive.

    

**Minor corrections** - Comments/Actions:

 - "This research and article has the potential to make a really interesting contribution to discussions on the gap between invention and commercialization, but currently it undersells itself by not drawing out more strongly the findings from the research and their implications for practice and theory".

Response: okay.

 - "This is a scientific paper and the language used should reflect it. Expressions such as 'go the extra mile', 'tap into', 'that concern with what will exist' and 'right up to' should be rewritten to a more formal language

Response: Sorted, a native proofreader revisit the paper.

 - "...what role does materiality play in design authorship? Going back to Schön’s notion of backtalk, how does the material that the designer is working on influence the design authorship? Building on this, the design authorship concept could also benefit from considering the temporal dimension. 

Response: Page 22, last paragraph.

 - "Questioning user-centricity is definitely a promising avenue, and then this discussion could be linked in the implications section back to entrepreneurs more broadly."   

Response: Page 23, first paragraph.    

**Minor corrections** - Comments/Actions:

 - "p. 4, “Design entrepreneurs fit within this description” <- how exactly? This could be expanded a bit more." 

Response: Eliminated.

 - "p. 4, “was in charged” <- d should be removed"

Response: Changed

 - "p. 4, not sure the section on “Design models” is necessary." 

Response: This section has been included to show the reader the connection between the stablished design models and the entrepreneurial milestones.

 - "p. 4, “mixed research team” <- not sure this is necessary if “mixed” in this context only means gender. Instead, maybe it would make sense to describe the authors’ positionality a bit more." 

Response: Changed. Explained in page 9 third paragraph.

 - "Since the study utilizes visuals and verbal accounts, it might be worthwhile to look into multimodal research (e.g. Höllerer et al., 2018; Kress & van Leeuwen, 2020)" 

Response: Constructivist Grounded Theory is a form of Multimodal research. Its emphasis is prioritising the integration of various data sources and data types.

 - "Notions of ethos could be strengthened by drawing on Fayard I.’s (2017) work." 

Response: We include this in Page 20

 - "Would love to see the design authorship concept visualized somehow to increase its chances of getting cited by future research."

Response: There is a video abstract of the D-Authorship https://youtu.be/F3sOyEqp3BI

 - "Think it would make sense to have a more nuanced discussion of designers and design authorship – graphic designers are not the same as fashion designers, and at the same time not every designer is trained to be an artistic designer (thinking of Bruno Munari here)."   

Response: In this research project, this is outside of the scope given the product based designer entrepreneurs.

 - "Flagging it here once more just in case, but how many of the informants were solo entrepreneurs and how many were part of a team?"

Response: All of our designer entrepreneurs had support and collaborators. But in the end they were the ones going through the whole process.

 - "Practical implications could be expanded as there are some very interesting points here! Garbuio *et al*.’s (2018) study on design cognition and entrepreneurship should be useful here." Response: We expanded the practical implications following the previous recommendations.  

 Thank you once again for such a meticulous review. 

 Best Regards,

 Dr Aldo Valencia

 Newcastle Business School, Northumbria University, UK.
